# Evaluation of endometrial receptivity in women with unexplained infertility by shear wave elastography

**DOI:** 10.1186/s13244-024-01646-3

**Published:** 2024-03-22

**Authors:** Zheng-ying Li, Lu Cai, Zhi-jun Zhang, Hai-rong Zou, Mei He, Mei-lin Qin, Hui Wang

**Affiliations:** Department of Ultrasound, University-Town Hospital of Chongqing Medical University, No. 55 University Middle Road, Shapingba District, Chongqing, 401331 China

**Keywords:** Endometrial receptivity, Endometrium, Elasticity, Shear wave elastography, Unexplained infertility

## Abstract

**Objectives:**

The endometrium of most unexplained infertility (UI) patients has been altered histologically. Shear wave elastography (SWE) is utilized to assess the signature of living tissue. This study aimed to explore the value of SWE in evaluating endometrial receptivity (ER) in UI patients.

**Methods:**

In total, 59 UI patients (UI group) and 52 normal control women (NC group) who received fertility consultation in our hospital were included between January 2022 and June 2023. We divided them into the late-proliferative phase of UI group (LPUI; *n* = 59), mid-secretory phase of UI group (MPUI; *n* = 41), late-proliferative phase of NC group (LPNC; *n* = 52), and mid-secretory phase of NC group (MPNC; *n* = 45). Transvaginal ultrasonography and SWE were performed during the LP and MP. Endometrial thickness (EMT), uterine artery pulsatility index (UA-PI), endometrial mean elasticity (E-mean), and mean shear wave velocities (SWV-mean) were measured.

**Results:**

There were significant differences in E-mean, SWV-mean, EMT, and UA-PI between the UI group and the NC group during both the LP and MP (*p* _MPNC vs MPUI_ < 0.05, *p* _LPNC vs LPUI_ < 0.05). E-mean and SWV-mean decreased with increasing EMT but increased with increasing UA-PI (*p* < 0.05). The most effective parameter for evaluating ER in UI patients is the E-mean (AUC = 0.89).

**Conclusions:**

UI patients exhibited thinner endometrium, increased endometrial stiffness, and poor endometrial blood perfusion. E-mean was the most effective parameter to evaluate ER in UI patients. The study preliminarily proved that SWE is a promising non-invasive tool for evaluating the condition of endometrium.

**Critical relevance statement:**

This study aimed to explore the significance of endometrial elasticity measured by SWE in evaluating patients with UI. The findings revealed a correlation between EMT, UA-PI, and E-mean. Endometrial elasticity can serve as an effective indicator for predicting ER.

**Key points:**

1. To explore the significance of endometrial elasticity in assessing patients with UI.

2. The endometrium of UI patient exhibited thinness, stiffness, and poor blood perfusion.

3. Endometrial elasticity serves as a valuable indicator for evaluating endometrial receptivity.

**Graphical Abstract:**

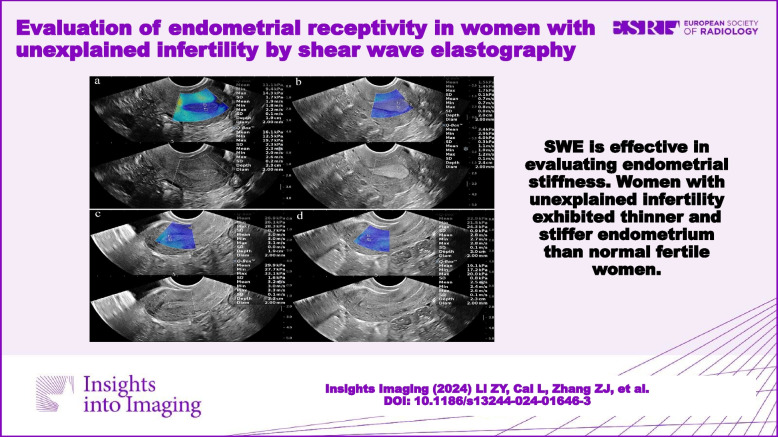

## Introduction

Unexplained infertility (UI) is a condition characterized by a couple’s inability to conceive for over a year, despite engaging in regular sexual intercourse, and no apparent causes for infertility have been identified through routine diagnostic evaluation [1]. Currently, approximately 10% to 30% of women with UI have an unknown etiology, with implantation failure being believed to be the primary cause [2]. Endometrial receptivity (ER) plays a significant role, accounting for as much as 50–60%. This refers to the condition of the endometrium being prepared to receive blastocysts [3, 4]. Transvaginal ultrasound is a preferred non-invasive method for assessing endometrial receptivity (ER). It can provide information about various aspects such as endometrial thickness (EMT), echo pattern, endometrial peristalsis, and endometrial and subendometrial blood perfusion [5, 6]. Previous studies have indicated that tissues exhibit different elasticity due to differences in cellular composition and content. Pathological changes in cells can significantly impact the elasticity and stiffness of the corresponding tissues [7, 8]. However, these aforementioned parameters that directly or indirectly reflect the function and morphology of the endometrium may not effectively reflect the characteristic of endometrial stiffness.

In recent studies, shear wave elastography (SWE) has been utilized to assess tissue elasticity and distinguish between normal and pathological tissue [9, 10]. SWE is an emerging ultrasound technology that measures shear wave velocity to quantify tissue elasticity [11]. It offers advantages such as non-invasiveness, real-time imaging, and repeatability. Currently, SWE is employed in gynecology primarily for diagnosing and differentiating uterine diseases. However, there is limited research on its application in evaluating the endometrium. Some investigators have applied SWE to assess the women after artificial abortion and found that endometrial stiffness increased with the increasing number of artificial abortions, which indicated potential endometrial damage that was not observed on conventional ultrasound [12].

In this study, we used shear wave elastography to evaluate the elasticity characteristics of the endometrium in patients with UI during the late-proliferative phase and mid-secretory phase. Our aim was to explore the value of endometrial stiffness in evaluating endometrial receptivity in patients with UI.

## Materials and methods

This retrospective study was conducted in accordance with the Declaration of Helsinki and approved by the Institutional Ethics Committee of University-Town Hospital of Chongqing Medical University (protocol code LL-202266).

### Subjects

In this retrospective study, we analyzed a total of 111 women who received fertility consultation or pre-pregnancy examination at University-Town Hospital of Chongqing Medical University between January 2022 and June 2023. The study included 59 cases of women with UI (UI group) and 52 cases of normal fertile women (NC group). Patients with UI were defined as those who had normal parameters based on routine diagnostic evaluation for infertility women, according to the WHO criteria. In addition, the normal fertile women in our study who have been proved clinical pregnancy within 3–6 months after undergoing this examination.

### Inclusion and exclusion criteria

The patients in the groups were females within the childbearing. They had a normal menstrual cycle of 26–32 days and normal morphology of the uterus and ovary. Hormonal drugs were not used, and no gynecological surgery had been performed for at least 2 months prior to the examination. Ovulation was confirmed through B-ultrasound. Exclusion criteria included women with uterine malformation, fallopian tube lesions, or a history of ovarian disease. Additionally, women with cardiopulmonary disease, abnormal liver and kidney function, or those receiving vasodilator therapy were excluded. Women whose male partners had abnormal semen analysis were also excluded.

**Fig. 1:**
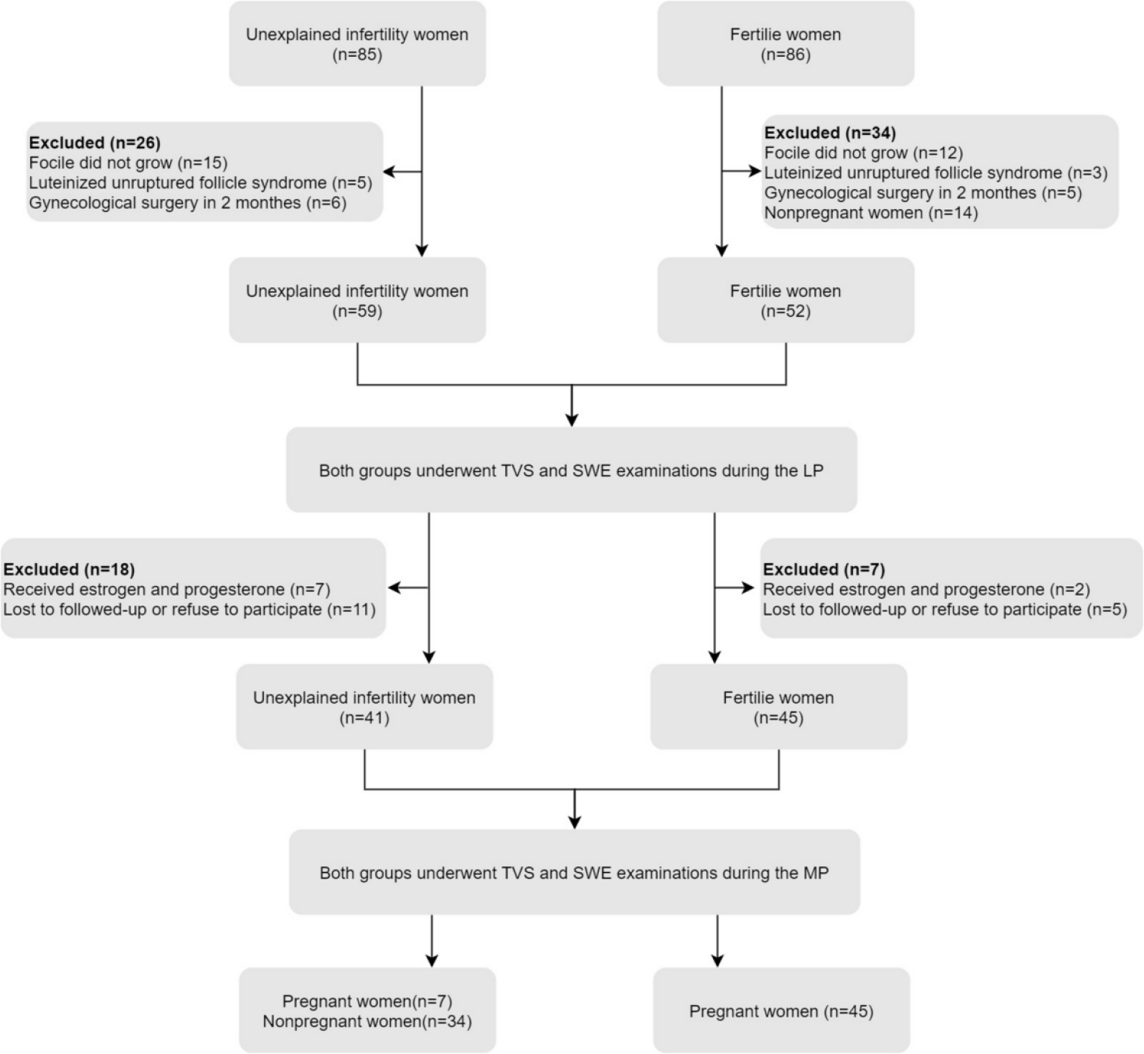
Flow chart of the study population. *TVS* transvaginal ultrasonography; *SWE* shear wave elastography; *LP* late-proliferative phase; *MP* mid-secretory phase

Women underwent hormonal and transvaginal ultrasound ovulation monitoring starting from the 10th day of their menstrual cycle, which was performed every other day to determine the day of ovulation. Conventional transvaginal ultrasonography (TVS) and SWE were performed during the late-proliferative phase (LP; ovulation phase; days 12–16) and mid-secretory phase (MP; window of implantation; 6–9 days after ovulation), respectively. However, during the MP, 7 UI patients and 2 normal women received estrogen and progesterone, and 11 UI patients and 5 normal women were lost to follow-up and therefore excluded. Finally, we divided the UI group (21–35 years old) into the LPUI group (59 cases) and the MPUI group (41 cases) based on the inspections conducted during the LP and MP. Similarly, the NC group (22–35 years old ) was divided into the LPNC group (52 cases) and the MPNC group (45 cases) (Fig. 1).

### TVS and SWE acquisition

Supersonic Imagine AixPlorer (SuperSonic, Aix-en-Provence, France) ultrasound device was equipped with a SE12-3 endocavitary probe (3–12 MHz), which is applicable for SWE. All TVS and SWE were performed by a sonographer with 5 years of experience in gynecologic ultrasonography. All patients’ informed consent was obtained before the examinations.

TVS and SWE examinations were performed with the women in the lithotomy position and with an empty bladder. The SE12-3 endocavitary probe placed in the posterior fornix of the vagina to obtain a two-dimensional sagittal section of the entire uterine cavity. Endometrial thickness (EMT) was measured at 2 cm from the bottom of the uterus in the largest sagittal section. Then, the endocavitary probe was rotated 90° to acquire a cross section of the cervix. Uterine artery pulsatility indexes were measured near the lateral-inferior margin of the uterine cervical junction in the right and left main uterine artery (Corr < 60°, SV < 2 mm).

**Fig. 2:**
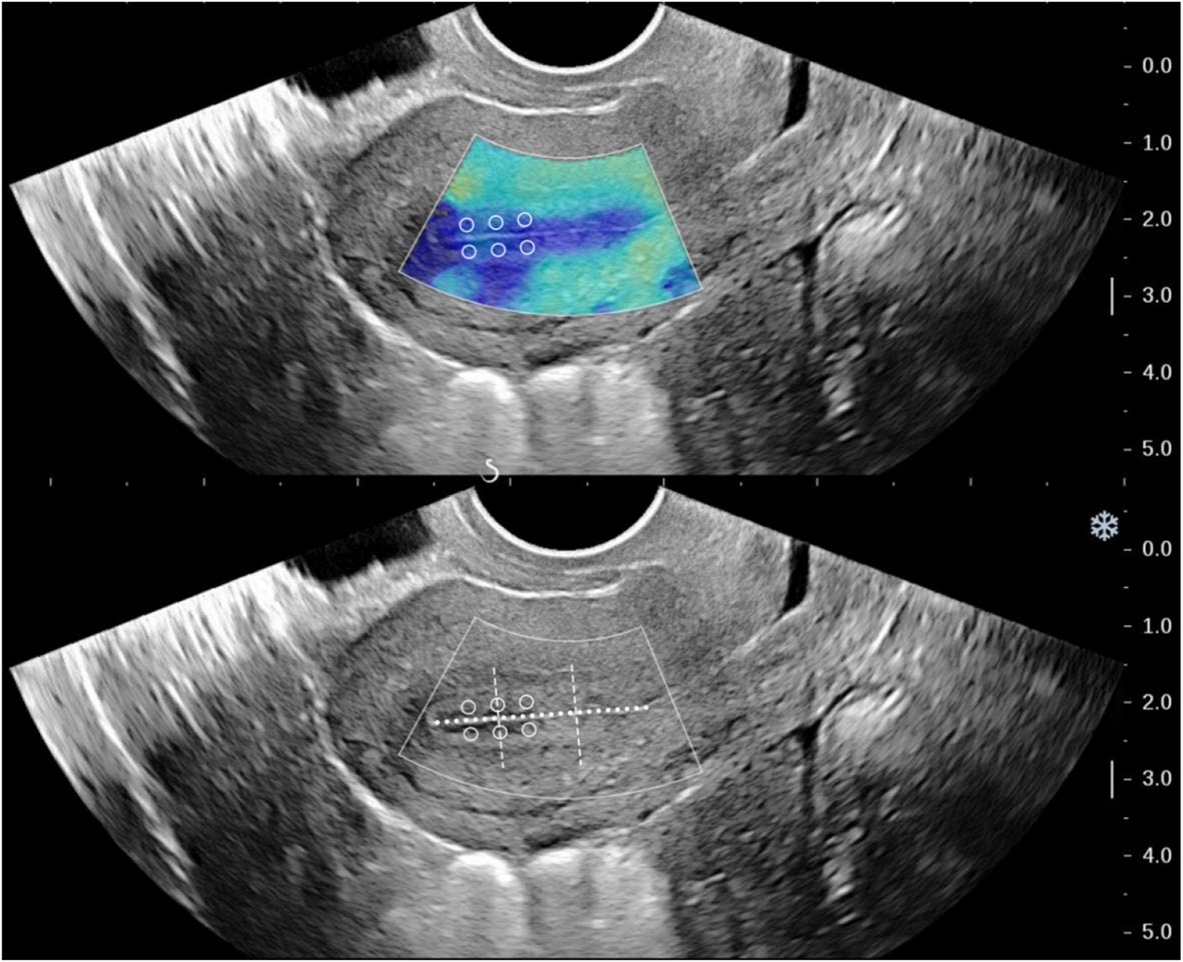
Example sonographic image of the region of interest (ROI) used for measuring endometrial elasticity using SWE. Notes: In a standard sagittal section of the uterus, the endometrium is completely covered by the white sampling frame; the white horizontal dotted line along the uterine cavity line is of the same length as the endometrium; the other two white dotted lines are vertical to the endometrial line and divide the endometrium into three segments; the white circles indicate the six sampling sites for SWE located in the middle and upper segments of the endometrium

Endometrial elasticity can be measured using SWE at the uterus in the largest sagittal section. To ensure accurate measurements, the sampling frame should completely wrap around the endometrium. The image should be frozen when it stabilizes, after resting for 3 s. The diameter of the region of interest (ROI) should be set to 2 mm, with the distance between the ROI and the probe surface ranging from 2 to 4 cm. In the standard sagittal section of the uterus, the midpoint between the myometrium-endometrial boundary and the uterine line is selected. Three points are chosen as regions of interest (ROI) along the upper middle segment of the anterior endometrial strip and the posterior endometrium, respectively, with each point spaced 3–5 mm apart (Fig. 2). The colors red, green, and blue represent high, medium, and low Young’s modulus, respectively. The Q-box system automatically calculates the average elastic modulus (E-mean) and shear wave velocity (SWV) of the endometrium in the ROI. All the above indicators were measured three times in the same part, and the average of these measurements was calculated.

### Clinical data

All relevant clinical data regarding the characteristics of the included population were collected (Table 1). And the basic hormone levels of all patient’s follicle-stimulating hormone (FSH), luteinizing hormone (LH), prolactin (PRL), estradiol (E2), progesterone (P), and testosterone (T) were recorded before ovulation (days 12–16). In this study, clinical pregnancy was specifically defined as the detection of a gestational sac in the uterus, containing an embryo with a confirmed heartbeat, as determined by ultrasound.

**Table 1:** Clinical data between normal control group and unexplained infertile group

Groups	Cases (*n*)	Age (years)	BMI (kg/m^2^)	Follicle-stimulating hormone (mIU/mL)	Luteinizing hormone (mIU/mL)	Estradiol (ng/mL)	Testosterone (ng/mL)	Prolactin (ng/mL)
NC	52	29.15 ± 3.53	19.85 ± 1.43	10.45 ± 3.95	35.46 ± 16.78	138.43 ± 70.02	0.32 ± 0.13	15.74 ± 6.14
UI	59	28.58 ± 3.32	19.41 ± 1.08	9.82 ± 3.70	36.42 ± 18.12	136.05 ± 61.26	0.35 ± 0.10	15.07 ± 5.74
z/t		0.887	-1.927	-0.860	-0.245	-0.009	-1.248	0.592
*p*		0.377^b^	0.054^a^	0.390^a^	0.806^a^	0.993^a^	0.212^a^	0.555^b^

*NC* Normal control, *UI* Unexplained infertility, *BMI* Body mass index

^a^Mann-Whitney *U* test

^b^Student’s test

*p* < 0.05, indicating statistical significance

### Statistical analysis

The measurement data were analyzed by the SPSS25.0 statistical software package, and the quantitative data were subjected to the Shapiro-Wilk (SW) normal distribution test. The data were presented as mean ± standard deviation, and we performed the *t*-test if it is in line with the normal distribution, while if not, a Mann-Whitney *U* test was conducted. Spearman rank correlation analysis was conducted to investigate the relationship between two periods of EMT, PI, and endometrial E-mean. Binary logistic regression was utilized to analyze the association between the window period uterine artery PI, EMT, endometrial E-mean, and patients with UI. The receiver operating characteristic (ROC) curve was plotted using the window period uterine artery PI, EMT, and endometrial E-mean, and the specificity, sensitivity, area under the ROC curve (AUC), and critical value were determined. The significance level was set at *α* = 0.05 (two-tailed).

## Result

### Population’s characteristics

The basic clinical data of patients with UI and normal women, showing that there were no significant differences in age, BMI, and basic hormone levels between the UI group and NC group (*p >* 0.05) (Table 1).

### TVS and SWE results

**Fig. 3:**
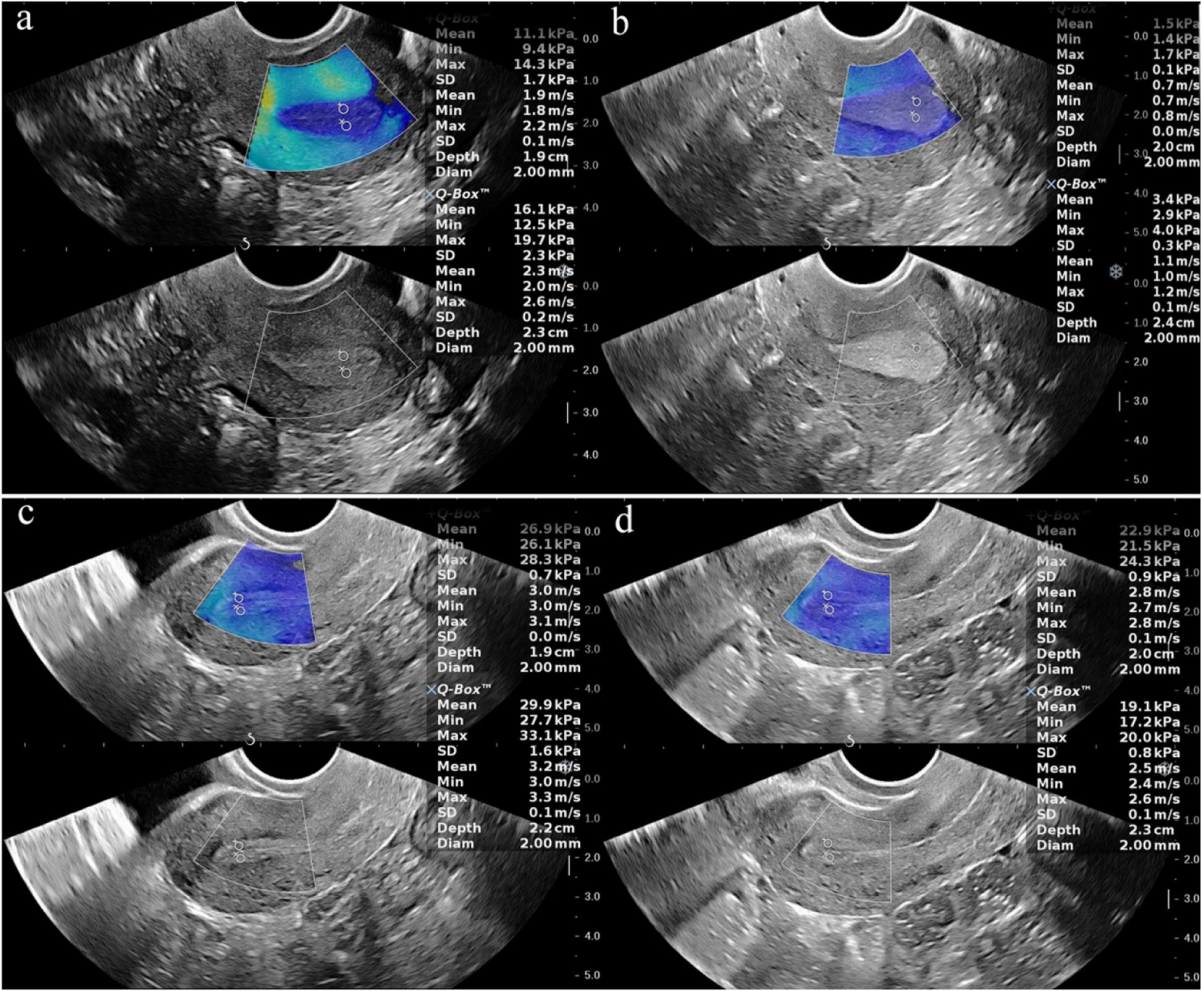
Representative images of a woman in the NC group during the LP (**a**) and MP (**b**). **a** In the LPNC group, the mean elasticity (E-mean) and mean shear wave velocity (SWV-mean) at the two ROIs of the upper endometrial segments were 11.1kPa and 16.1 kPa and 1.9 m/s and 2.3 m/s, respectively. **b** In the MPNC group, the E-mean and SWV-mean at the two ROIs of the upper endometrial segments were 1.5 kPa and 3.4 kPa and 0.7 m/s and 1.1 m/s, respectively. Representative images of a woman in the UI group during the LP (**c**) and MP (**d**). **c** In the LPUI group, the E-mean and SWV-mean at the two ROIs of the upper endometrial segments were 26.9 kPa and 29.9 kPa and 3.0 m/s and 3.2 m/s, respectively. **d** In the MPUI group, the E-mean and SWV-mean at the two ROIs of the upper endometrial segments were 22.9 kPa and 19.1 kPa and 2.8 m/s and 2.5 m/s, respectively

**Fig. 4:**
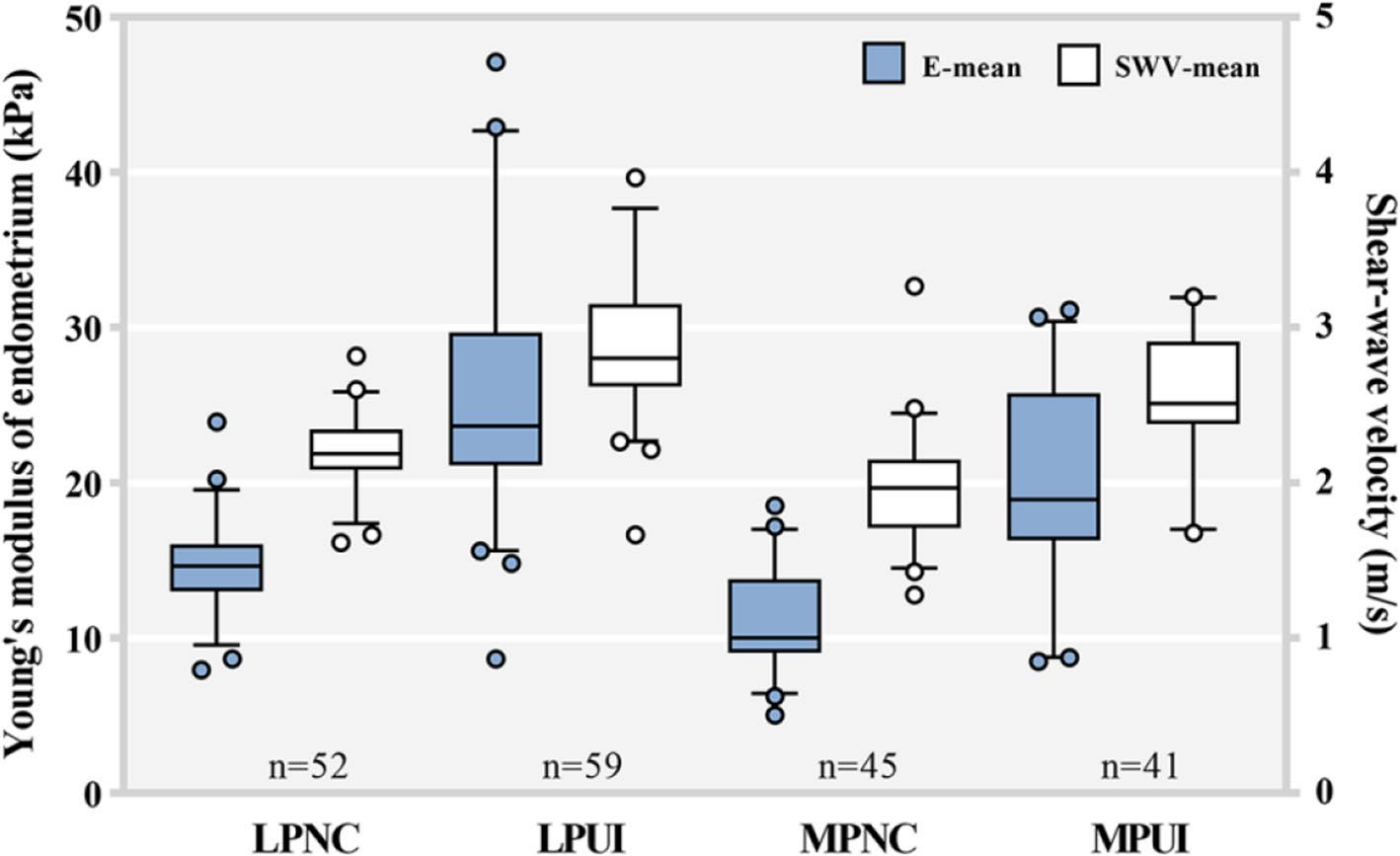
Box and whisker plot showing the average values of Young’s modulus (E-mean, represented by the blue box) and shear wave velocity (SWV, represented by the white box) for both normal fertile women and patients with UI during the LP and MP. Box plots indicate interquartile ranges (in kPa and m/s)

**Fig. 5:**
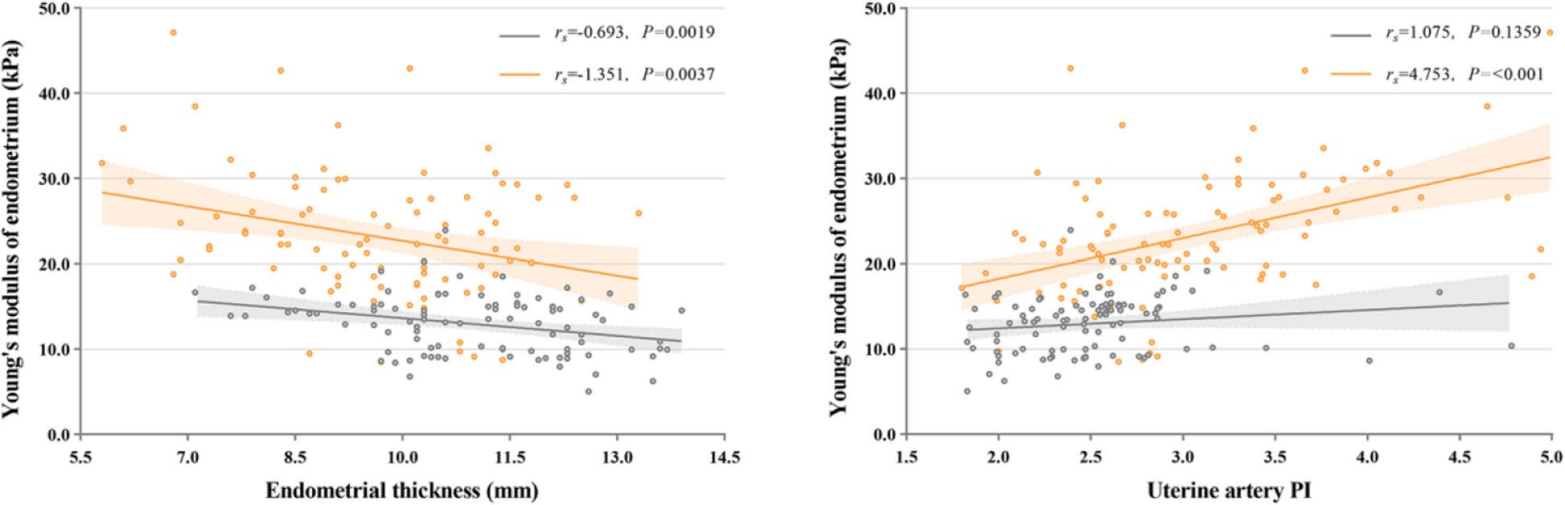
Analysis of the correlation between endometrial thickness (mm) and the average Young’s modulus (kPa) in UI group (orange circles and lines) compared with the NC group (gray circles and lines). Mean (thick lines) and 95% confidence interval (thin lines) values are shown. Analysis of the correlation between mean UA-PI of bilateral uterine arteries and the average Young’s modulus (kPa) in UI group (orange circles and lines) compared with the NC group (gray circles and lines). Mean (thick lines) and 95% confidence interval (thin lines) values are shown

The data in Table 1 demonstrate the EMT of the UI group was thinner than that of the NC group during both the late-proliferative phase (LP) and the mid-secretory phase (MP) (*p* _LPNC vs MPNC_ < 0.05, *p* _LPUI vs MPUI_ > 0.05). Moreover, the EMT of both the NC group and the UI group was significantly higher in the MP compared to the LP (*p* _LPNC vs LPUI_ < 0.05, *p* _MPNC vs MPUI_ < 0.05). Both the NC group and UI group showed lower UA-PI PI during the MP compared to the LP (*p* _LPNC vs MPNC_ < 0.05, *p* _LPUI vs MPUI_ > 0.05). The UA-PI in the UI group was significantly higher than the NC group in both periods (*p* _LPNC vs LPUI_ < 0.05, *p* _MPNC vs MPUI_ < 0.05). The E-mean and SWV were significantly lower during the MP than the LP in both groups (*p* _LPNC vs LPUI_ < 0.05, *p* _MPNC vs MPUI_ < 0.05) ( Fig. 3), while they were significantly higher in the UI group compared to the NC group in both periods (*p* _LPNC vs LPUI_ < 0.05, *p* _MPNC vs MPUI_ < 0.05) (Table 2; Fig. 4). In both the NC group and UI group, there was a correlation between the EMT and UA-PI with the E-mean. Specifically, E-mean decreased with increasing EMT but increased with the increasing UA-PI (*p* < 0.05) (Fig. 5).

**Table 2:** Parameters of NC and UI group during the late-proliferative and mid-secretory phase

Groups	Cases (*n*)	EMT (mm)	UA-PI	E-mean (kPa)	SWV-mean (m/s)	Days after ovulation (days)
NC						
LPNC	52	10.37 ± 1.46	2.64 ± 0.52	14.40 ± 2.92	2.18 ± 0.24	
MPNC	45	11.68 ± 1.27	2.35 ± 0.38	10.18 ± 3.29	1.79 ± 0.30	7.62 ± 0.81
UI						
LPUI	59	9.35 ± 1.63	3.14 ± 0.63	25.57 ± 7.06	2.88 ± 0.41	
MPUI	41	10.03 ± 1.35	2.90 ± 0.75	21.51 ± 7.63	2.61 ± 0.51	7.51 ± 0.64
*p* _LPNC vs LPUI_		0.001^b^	< 0.001^a^	< 0.001^a^	< 0.001^a^	
*p* _MPNC vs MPUI_		< 0.001^b^	< 0.001^a^	< 0.001^a^	< 0.001^a^	0.601^a^
*p* _LPNC vs MPNC_		< 0.001^b^	< 0.001^a^	< 0.001^a^	< 0.001^a^	
*p* _LPUI vs MPUI_		0.786^b^	0.531^a^	0.845^a^	0.753^a^	

*NC* Normal control, *LPNC* Late-proliferative phase of normal control, *MPNC* Mid-secretory phase of normal control, *UI* Unexplained infertility, *LPUI* Late-proliferative phase of unexplained infertility, *MPUI* Mid-secretory phase of unexplained infertility, *EMT* Endometrial thickness, *UA-PI* Uterine artery PI, *E-mean* Mean elasticity (or mean Young’s modulus), *SWV-mean* Mean shear wave velocities

^a^Mann-Whitney U test

^b^Student’s test

*p* < 0.05, indicating statistical significance

### Pregnancy outcomes of unexplained infertility and ultrasound assessment

Seven women with UI had experienced a clinical pregnancy, with the visualization of embryos and the presence of heartbeats confirmed through ultrasound. The ultrasonic parameters of endometrial receptivity (ER) during MP for both pregnant and non-pregnant women in patients with UI. The study found no significant difference in EMT and UA-PI between the two groups (*p* > 0.05). However, the E-mean and SWV-mean of non-pregnant women were significantly higher than those of pregnant women in patients with UI (*p* < 0.05) (Table 3).

**Table 3:** Ultrasound parameters of endometrial receptivity during mid-secretory phase for both pregnant and nonpregnant women in patients with UI

Pregnancy outcome of UI patients	Cases (*n*)	Endometrial thickness (mm)	UA-PI	E-mean (kPa)	VS-mean (m/s)
Nonpregnant	34	9.97 ± 1.44	2.92 ± 0.82	24.03 ± 5.77	2.90 ± 0.65
Pregnant	7	10.33 ± 0.75	2.80 ± 0.26	17.83 ± 7.15	2.39 ± 0.52
***t/z***		-0.635	-0.450	2.486	-2.201
***p***		0.529^b^	0.652^a^	0.017^b^	0.028^a^

^a^Mann-Whitney *U* test

^b^Student’s test

*p* < 0.05, indicating statistical significance

**Fig. 6:**
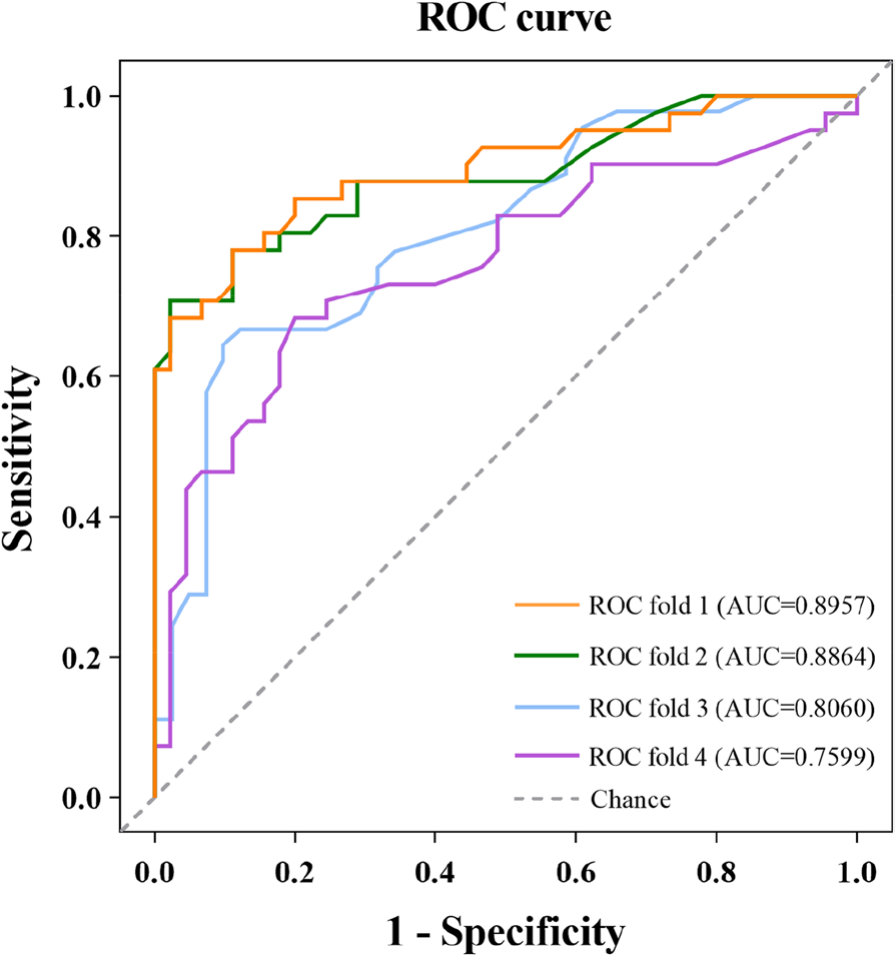
ROC curves of the predictive value for endometrial receptivity during mid-secretory phase in women with UI. ROC fold 1: average Young’s modulus; ROC fold 2: mean shear wave velocity; ROC fold 3: endometrial thickness; ROC fold 4: uterine artery PI

Figure 6 reveals that the most effective parameter for evaluating endometrial receptivity during the mid-secretory phase in UI patients is the E-mean, with an area under the curve (AUC) of 0.896. The most discriminative threshold value for binarizing E-mean was 14.75 kPa, resulting in a sensitivity of 78.0% and a specificity of 88.9%. In contrast, the UA-PI had a poor value with an AUC of 0.760. The most discriminative threshold value for binarizing UA-PI was 2.54, resulting in a sensitivity of 68.3% and a specificity of 80.0%.

## Discussion

Several studies have shown that patients with UI exhibit poor endometrial receptivity during the mid-secretory phase [13–15]. Additionally, Bergeron et al. found that some disturbances in the secretory phase of endometrium can be traced back to disturbances in the proliferative phase [16]. And the endometrium of UI patients has been altered histologically [17]. Conventional ultrasonography may not be able to detect this change, but SWE can provide a quantitative evaluation of the texture, softness, and stiffness of the tissue in the region of interest [7]. Therefore, in this study, we observed the changes of EMT, endometrial elasticity, and uterine artery PI in patients with UI during the late-proliferative phase and the mid-secretory phase; setting out the value of endometrial elasticity as a way to assess the endometrial receptivity in women with UI.

In this study, endometrial stiffness was significantly lower during mid-secretory phase in both the NC and UI groups than that during late-proliferative phase. This finding is consistent with the findings of Du et al. [18], who also reported that the endometrium is softer during the secretory phase compared to the proliferative phase. The functional layer of the endometrium undergoes hormone-dependent morphological and physiological changes, leading to endometrial thickening during the proliferative and secretory phases [19]. Our results also support this. The process of endometrial cell proliferation and extracellular matrix remodeling mainly occurs during the proliferative phase [20]. During this phase, the stromal cells take on a fibroblast-like appearance, then undergo decidualization in the mid-secretory phase [21]. Based on these results, we suspected that it may be due to an increased number of endometrial glands and the decidualization of stromal cells, which is to prepare for implantation. In addition, both the NC group and the UI group exhibited lower UA-PI during the MP compared to the LP. Consistent with the previous findings, the UA-PI varying with the menstrual cycle, being lower in the mid-secretory phase, indicates increased blood perfusion in the endometrium [22, 23]. It suggested that better endometrial blood perfusion during the MP is more favorable for embryo implantation.

We found that the E-mean and SWV-mean of UI patients were higher than those of normal women in both periods; as the SWV-mean of the endometrium increased, the E-mean also increased. This suggests that the endometrial tissue stiffness is higher in patients with UI. Özdem Karaoğlan et al. conducted secretory endometrium biopsies on UI patients and observed that the endometrial glands were sparser and less curved, while the stromal components were denser compared to the fertile group [24]. The endometrial stroma contains abundant fibroblasts that secrete collagen fibers [25]. Some researchers observed that the more glandular components are associated with a softer endometrium [10]. It is hypothesized that the higher endometrium stiffness in patients with UI could be attributed to the decreased glandular components of the endometrium and inadequate decidualization of the dense matrix in UI patients. Additionally, our study revealed that the E-mean is a reliable parameter for assessing endometrial receptivity in UI patients. Therefore, the evaluation of endometrial stiffness using SWE can provide a valuable clinical index for assessing ER. In cases where infertile patients exhibit abnormal endometrial elasticity, it is important to consider implementing appropriate treatment strategies to improve implantation.

Studies have shown that the thickness of the endometrium is critical for embryo implantation. These studies have identified that EMT is reliable parameter for evaluating ER and accurately predicting the outcome of pregnancy [26, 27]. In this study, it was observed that the EMT in the UI group consistently remained thinner than the NC group in both periods. It shows that thin EMT may be one of the reasons for implantation failure in patients with UI. The mechanism may be that patients with a thin endometrium have a reduced functional layer, causing the embryo to primarily implant in the basal layer. The higher oxygen concentration near the spiral artery in the basal layer may not be favorable for embryo implantation [28]. We also found that the UA-PI in the MPUI group was significantly higher than that in the MPNC group, implying poor endometrial blood perfusion in UI patients during the implantation window period, which is similar with the research results of Smart and El-Mazny et al. [29, 30]. In addition, the UA-PI of the LPUI group was also significantly higher than that of the LPNC group, suggesting that the decrease in endometrial blood perfusion in UI patients may had occurred in the proliferative phase.

Recent research shows that poor endometrial blood perfusion during proliferation may further lead to endometrial dysplasia and failure of embryo implantation [31]. Our analysis revealed a correlation between the EMT and UA-PI with the E-mean in both UI group and NC group, indicating that both EMT and UA-PI can impact endometrial stiffness. Specifically, when the endometrium is affected by dysplasia or poor blood perfusion, it tends to exhibit increased stiffness. This elevated stiffness can subsequently contribute to the failure of embryo implantation. However, we found in the follow-up that during MP, there was no statistically significant difference in EMT and UA-PI between pregnant women and non-pregnant women in the UI group. And we observed that the E-mean and SWV of non-pregnant women were significantly higher than those of pregnant women. Our findings indicated that non-pregnant women with UI have higher endometrial stiffness compared to pregnant women. We suspected that there may be underlying pathological changes in the endometrium of certain patients with UI, which cannot be detected through conventional ultrasound measurement of EMT and uterine artery, while SWE could detect disturbances preceding changes in endometrial thickness and perfusion.

This study has several limitations. Firstly, the sample size of the study is relatively small due to the special UI population. Conducting a future multi-center study with a larger sample size would be necessary. Secondly, since most patients were unwilling to undergo endometrial biopsy, the histopathological endometrium was not taken into account in this study. However, we will continue to follow-up more pathological results in the later stage to analyze the correlation between the pathological characteristics and the hardness of the endometrium. Finally, it is notable that the uterus is a three-dimensional organ, but in this study, elastography of the endometrium was performed in two-dimensional slices. Therefore, the selected ROI may not fully represent the overall endometrium. Nevertheless, in this study, multiple ROIs were sampled in the upper middle segment of the endometrium, where blastocysts are most likely to implant, in order to minimize this effect.

## Conclusion

In conclusion, SWE is effective in quantitatively evaluating the endometrial characteristics of UI patients. The endometrial stiffness can provide valuable information about assessing the ability of the endometrium to accept embryo implantation. This preliminary study suggests that SWE has the potential to predict endometrial receptivity, which warrants further investigation in large-scale studies.

## Data Availability

The datasets used and/or analyzed during the current study are available from the corresponding author on reasonable request.

## Abbreviations

E-mean: Mean endometrial elasticity
EMT: Endometrial thickness
ER: Endometrial receptivity
LP: Late-proliferative phase
MP: Mid-secretory phase
NC: Normal control
SWE: Shear wave elastography
SWV-mean: Mean shear wave velocities
TVS: Transvaginal ultrasonography
UA-PI: Uterine artery PI
UI: Unexplained infertility
